# Plant-Produced Asialo-Erythropoietin Restores Pancreatic Beta-Cell Function by Suppressing Mammalian Sterile-20-like Kinase (MST1) and Caspase-3 Activation

**DOI:** 10.3389/fphar.2017.00208

**Published:** 2017-04-19

**Authors:** Elena Arthur, Farooqahmed S. Kittur, Yuan Lin, Chiu-Yueh Hung, David C. Sane, Jiahua Xie

**Affiliations:** ^1^Department of Pharmaceutical Sciences, Biomanufacturing Research Institute and Technology Enterprise, North Carolina Central University, DurhamNC, USA; ^2^School of Basic Medical Sciences, Ningxia Medical UniversityYinchuan, China; ^3^Carilion Clinic and Virginia Tech Carilion School of Medicine, RoanokeVA, USA

**Keywords:** pancreatic beta-cell death, insulin secretion, MST1, asialo-rhuEPO, cytoprotection

## Abstract

Pancreatic beta-cell death adversely contributes to the progression of both type I and II diabetes by undermining beta-cell mass and subsequently diminishing endogenous insulin production. Therapeutics to impede or even reverse the apoptosis and dysfunction of beta-cells are urgently needed. Asialo-rhuEPO, an enzymatically desialylated form of recombinant human erythropoietin (rhuEPO), has been shown to have cardioprotective and neuroprotective functions but with no adverse effects like that of sialylated rhuEPO. Heretofore, the anti-apoptotic effect of asialo-rhuEPO on pancreatic beta-cells has not been reported. In the current study, we investigated the cytoprotective properties of plant-produced asialo-rhuEPO (asialo-rhuEPO^P^) against staurosporine-induced cell death in the pancreatic beta-cell line RIN-m5F. Our results showed that 60 IU/ml asialo-rhuEPO^P^ provided 41% cytoprotection while 60 IU/ml rhuEPO yielded no effect. Western blotting results showed that asialo-rhuEPO^P^ treatment inhibited both MST1 and caspase-3 activation with the retention of PDX1 and insulin levels close to untreated control cells. Our study provides the first evidence indicating that asialo-rhuEPO^P^-mediated protection involves the reduction of MST1 activation, which is considered a key mediator of apoptotic signaling in beta-cells. Considering the many advantages its plant-based expression, asialo-rhuEPO^P^ could be potentially developed as a novel and inexpensive agent to treat or prevent diabetes after further performing studies in cell-based and animal models of diabetes.

## Introduction

Diabetes has evolved into a high priority global epidemic (America’s Biopharmaceutical Research Companies, 2014). The loss of insulin producing pancreatic beta-cells resulting from apoptotic cell death is a major problem in all forms of diabetes mellitus (Mathis et al., 2001; Rhodes, 2005). Beta-cells are especially sensitive to multiple physiological and pathological stressors, which lead to cell death because of their low expression of several genes encoding antioxidant enzymes critical for cell survival (Lenzen et al., 1996). Currently, there are no effective therapeutics to prevent the decline in functional beta-cell mass (Ardestani et al., 2014). The discovery of new therapeutics, directly targeting the apoptotic process to impede or even reverse beta-cell apoptosis and dysfunction, is urgently needed.

**FIGURE 1:**
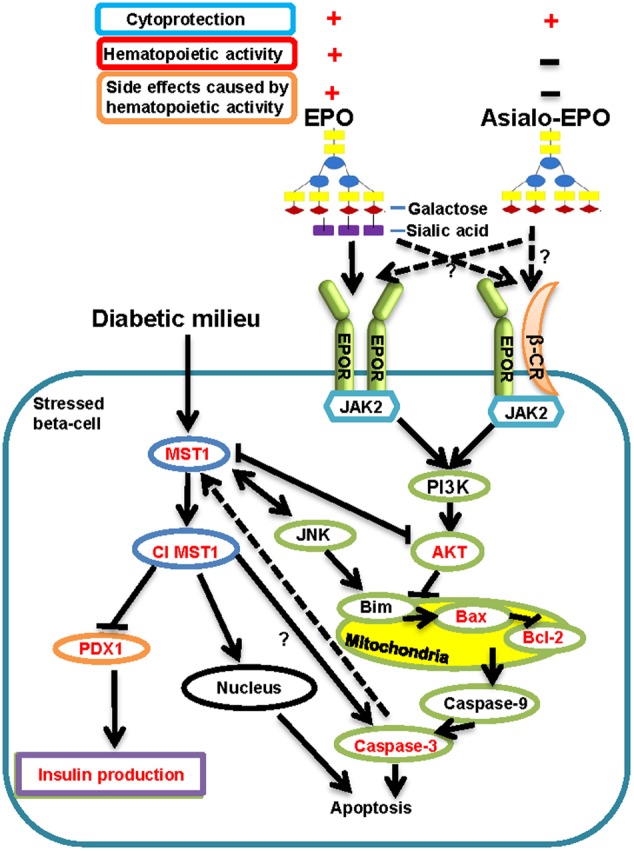
**Current view on how diabetic stimuli lead to activation of MST1 causing apoptosis and beta-cell dysfunction adopted from Ardestani et al. (2014), and how EPO and asialo-EPO display their cytoprotective functions via potentially binding to homodimeric EPOR or EPOR and β-CR heterodimeric receptor summarized from Brines and Cerami (2005, 2008).** Cl MST1: Cleaved MST1.

Recently, MST1, a ubiquitously expressed serine/threonine kinase, has been proven to be a novel pro-apoptotic kinase and key mediator of apoptotic signaling in beta-cells causing their dysfunction (Ardestani et al., 2014). MST1 is a component of the Hippo signaling pathway, which is responsible for regulating multiple cellular processes such as morphogenesis, proliferation, stress response and apoptosis (Ling et al., 2008; Avruch et al., 2012). Under diabetogenic conditions, MST1 is strongly activated in beta-cells triggering apoptosis (Ardestani et al., 2014). In the apoptosis signaling pathway, MST1 acts both as an activator as well as a target of caspase-3 (**Figure 1**) (Kakeya et al., 1998; Lee et al., 1998; Ardestani et al., 2014). The latter is also a major player of apoptosis (Ruest et al., 2002). Additionally, activated MST1 can directly phosphorylate PDX1, a key transcription factor involved in beta-cell development and function (Johnson et al., 2003). It has been shown that MST1-mediated phosphorylation of PDX1 leads to its ubiquitination and degradation (Ardestani et al., 2014). Down regulation of PDX1 affects beta-cell function and survival, resulting in impaired insulin production and secretion (Brissova et al., 2002; Johnson et al., 2003). Hence, MST1 has been suggested as a target for the discovery of new drugs for diabetes because of its critical role in beta-cell apoptosis and dysfunction (Ardestani et al., 2014). However, no agent has been identified yet to counteract the apoptotic activity of MST1 for the purpose of promoting beta-cell survival.

More than 180 medicines are being developed by biopharmaceutical companies to combat the rapidly rising global diabetes epidemic and its related conditions (America’s Biopharmaceutical Research Companies, 2014). Recently, rhuEPO, a glycoprotein hormone that regulates RBC production, has been shown to protect pancreatic beta-cells in animal models of diabetes (Choi et al., 2010; Chen et al., 2015) and diabetic nephropathy (Bianchi et al., 2004). Preclinical studies have shown that rhuEPO and its derivatives display remarkable anti-apoptosis and cytoprotection against damage triggered by stressors or cytotoxic agents in the brain, the heart, the kidneys and the liver (Brines et al., 2000; Calvillo et al., 2003; Vesey et al., 2004; Jelkmann, 2005; Jelkmann et al., 2009). Despite these exceptional tissue-protective activities of rhuEPO in mammalian cell and animal models, its therapeutic application for cytoprotection is hampered by the increased risk of thrombotic events resulting from its hematopoietic activity (Bennett et al., 2008). Furthermore, the cytoprotective doses of EPO are much higher than those required for stimulation of erythropoiesis (Brines and Cerami, 2008; Maiese, 2015) and its hematopoietic activity at these high doses can stimulate mass production of red blood cells causing adverse effects. Therefore, cytoprotective EPO derivatives lacking hematopoietic activity are highly desired.

Asialo-erythropoietin (**Figure 1**), an EPO derivative lacking sialic acid residues with a very short half-life in the circulation system, is devoid of hematopoietic activity (Erbayraktar et al., 2003). It was reported to exhibit excellent neuroprotective and renoprotective activities as rhuEPO but with no hematopoietic activity related side effects like thrombosis (Erbayraktar et al., 2003; Okada et al., 2007). However, the cytoprotective function of asialo-rhuEPO has not been tested in pancreatic beta-cells. We have developed a plant-based expression system to produce asialo-rhuEPO by stably co-expressing *EPO* and *GalT* genes in tobacco plants (Kittur et al., 2012, 2013). Using plant-based expression system can be expected to solve the issues that are associated with cost and large scale production from expensive rhuEPO^M^ for its production. We also demonstrated that asialo-rhuEPO^P^ possesses better cytoprotective effect than rhuEPO^M^ in protecting neuronal-like mouse neuroblastoma cells (Kittur et al., 2013) and murine HL-1 cardiomyocytes (F. Kittur et al., unpublished data) from STS-induced cell death. In these studies, we discovered that asialo-rhuEPO^P^ protects the above cells by down-regulating mitochondrial apoptotic pathways. Based on these results, and the fact that loss of beta cell mass occurs as a result of apoptosis, we reasoned that asialo-rhuEPO^P^ must also protect pancreatic beta-cells. Therefore, we investigated the protective effects of asialo-rhuEPO^P^ toward pancreatic beta-cells and insulin secretion. Since MST1 is one of the key players in beta-cell apoptosis and dysfunction, we also investigated whether asialo-rhuEPO^P^ has any effect on MST1 activation and PDX1 levels.

In the present study, we followed our previous approach (Kittur et al., 2013; F. Kittur et al., unpublished data) and used STS-induced apoptosis in the pancreatic beta-cell as a model to study the cytoprotective effects of asialo-rhuEPO^P^ and determine the involvement of MST1 in asialo-rhuEPO^P^-mediated cytoprotection in beta-cells. Our study revealed that asialo-rhuEPO^P^ protects pancreatic beta-cells from chemically induced apoptosis by preventing both MST1 and caspase-3 activation with the retention of PDX1 and insulin levels similar to untreated control cells.

## Materials and Methods

### Cell Culture and Cytotoxicity Assay

To test the cytoprotective effects of asialo-rhuEPO^P^ on pancreatic beta-cells, the cell line RIN-m5F (rat pancreatic β-cells, ATCC^®^ #: CRL 11605^TM^) was used. The RIN-m5F cells were cultured in RPMI 1640 medium (ATCC, Manassas, VA, USA) supplemented with 10% fetal bovine serum, 100 IU/ml penicillin, and 0.1 mg/ml streptomycin (Thermo Fisher Scientific, Rockford, IL, USA) in an incubator, and maintained at 37°C and 5% CO_2_. Cytotoxicity was assayed by measuring the amount of LDH released into the culture medium using a non-radioactive Cytotoxicity Detection Kit (Roche, Indianapolis, IN, USA).

### Determination of the EC_50_ Value of STS in RIN-m5F Cells

Staurosporine was used to induce apoptosis in the RIN-m5F cells. The 1 mM STS stock solution was purchased from Sigma–Aldrich (St. Louis, MO, USA). To determine the EC_50_ of STS, the concentration range and duration of treatment was adopted from Kittur et al. (2013). RIN-m5F cells were plated on a 96-well plate at a density of ∼1.5 × 10^5^ cells/well. At 80% confluence, cells were treated with 0, 0.1, 0.2, 0.3, 0.4, 0.5, and 0.6 μM STS. After 24 h treatment, the toxicity was determined using the LDH assay kit according to the manufacturer’s protocol. Each treatment was performed in six wells representing six replicates; and the experiment was repeated three times. The average percentage of cytotoxicity in three experiments was used to determine the EC_50_.

### Protective Effects of Asialo-rhuEPO^P^ on RIN-m5F Cells

To evaluate the protective effect, purified asialo-rhuEPO^P^ (Kittur et al., 2015) from our previously created tobacco transgenic line A56-5 (Kittur et al., 2013) was used to study its ability to protect RIN-m5F cells against STS-induced apoptosis. RIN-m5F cells were plated on a 96-well plate at a density of ∼1.5 × 10^5^ cells/well until 80% confluence. Then cells were treated with 0.123 μM STS alone, or 0.123 μM STS simultaneously with 20, 40, 60, 80, or 100 IU/ml purified asialo-rhuEPO^P^ in PBS containing 0.1% BSA for 24 h. As a vehicle control, same volume of PBS containing 0.1% BSA was added to the medium. For the STS only treatment, 0.123 μM STS in PBS containing 0.1% BSA was included in the medium. The number of asialo-rhuEPO^P^ units was calculated from protein concentration as described by Erbayraktar et al. (2003). LDH assay was used as mentioned above to determine cytotoxicity. To compare the cytoprotective effects of asialo-rhuEPO^P^ to regular rhuEPO^M^ purchased from R&D Systems (Minneapolis, MN, USA), cells were treated with 0.123 μM STS together with either 60 IU/ml asialo-rhuEPO^P^ or rhuEPO^M^ for 24 h. Vehicle control, STS alone treatment and cytotoxicity assay were set up as aforementioned. Each test was performed with six wells representing six replicates; and all tests were independently repeated four times as four biological replicates. The average of four replicates was used in the final calculations to compute cytotoxicity.

### Western Blotting

For western blot analysis, cells for each treatment or vehicle control were cultured at a density of 2.0 × 10^6^ in T-25 flasks until they reached 70% confluence. To monitor the activation of MST1 and caspase-3 under STS-induced toxic conditions, cells were treated with 0, 0.1, 0.2, 0.3, 0.4, 0.5, and 0.6 μM STS for 24 h. After that, both treated and control cells were collected and cell pellets were extracted with M-PER mammalian extraction reagent (Thermo Fisher Scientific, Rockford, IL, USA) to obtain total proteins. To investigate the cytoprotective mechanism of asialo-rhuEPO^P^, cells were also cultured at a density of 2.0 × 10^6^ in T-25 flasks and incubated at 37°C in 5% CO_2_ until they reached 70% confluence. Then cells in each flask were treated with 0.123 μM STS alone, or 0.123 μM STS simultaneously with 60 IU/ml purified asialo-rhuEPO^P^ or rhuEPO^M^. The same volume of PBS containing 0.1% BSA was added to the medium as a vehicle control. After 24 h of treatment, total proteins were extracted as previously described for the detection of MST1, caspase-3, pAKT/AKT, Bcl-2, Bax and PDX1. The experiment was repeated twice.

Protein separation and transfer were performed as described previously (Kittur et al., 2013). Membranes were blocked with 5% BSA in PBST for 1 h followed by overnight incubation at 4°C with the following primary antibodies: anti-caspase-3, anti-MST1, anti-pAKT, anti-AKT, anti-Bcl-2, anti-Bax, anti-PDX1, anti-β-tubulin (1:1000) (Cell Signaling, Danvers, MA, USA), and beta-actin (2 μg/μl) (Sigma–Aldrich, St. Louis, MO, USA). Following incubation and washing with PBST, blots were incubated with 1:2500 diluted HRP-conjugated secondary antibody for 1 h at room temperature. SuperSignal^®^ West Pico Chemiluminescent substrate (Thermo Fisher Scientific, Rockford, IL, USA) was used to detect protein bands.

### Insulin Measurement

Secreted insulin levels were measured in the spent media from the above four treatments (vehicle control, 0.123 μM STS alone, or 0.123 μM STS simultaneously with 60 IU/ml purified asialo-rhuEPO^P^ or rhuEPO^M^) for investigating how protective effects of asialo-rhuEPO^P^ are translated in insulin secretion. An ultrasensitive rat insulin ELISA kit (Crystal Chem, Chicago, IL, USA) was used to detect insulin following the manufacturer’s protocol. Briefly, spent media were collected and stored at –20°C until further analysis by ELISA. Five microliter each was used. The measurement was repeated three times. The average of two batches of experiments was used to calculate the secreted insulin levels.

### Statistical Analysis

All results were presented as the mean ± SD or SE where appropriate. Statistical significance was analyzed using One-way ANOVA and with student’s *t*-tests for pairwise mean comparison (*p* < 0.05).

## Results

### Establishment of STS-induced Apoptosis Pancreatic Beta-cell Model

Staurosporine is a common inducer of apoptosis in many cell types, and STS-induced apoptosis is believed to be triggered via activation of caspase-3 (Jacobson et al., 1996). MST1 has been recently discovered as a key mediator of apoptotic signaling in pancreatic beta-cells (Ardestani et al., 2014). In order to study the anti-apoptotic effects of asialo-rhuEPO^P^ on pancreatic beta-cells, we first established a STS-induced apoptosis beta-cell model by measuring induced cytotoxicity, and subsequently analyzing the activation of two apoptotic hallmarks caspase-3 and MST1 to confirm the established model.

**FIGURE 2:**
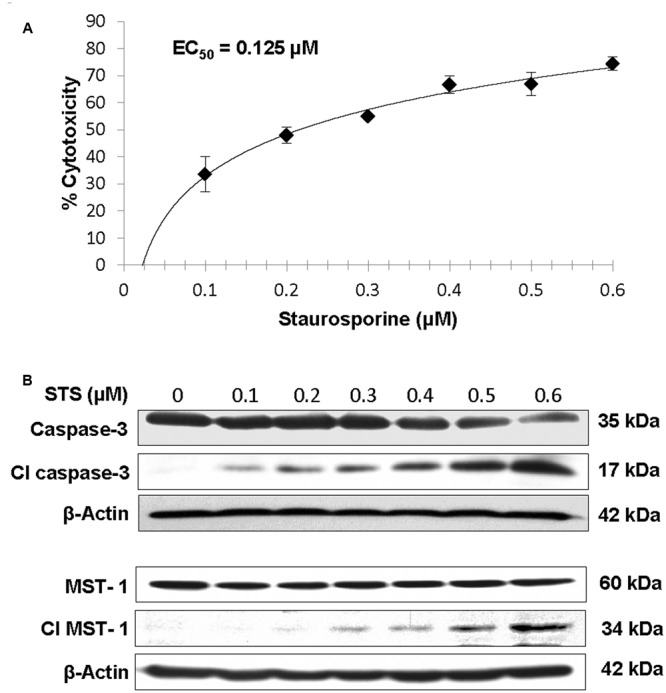
**Dose-dependent increase in cytotoxicity (A)**, caspase-3 activation and MST1 activation **(B)** in RIN-m5F cells incubated with 0–0.6 μM STS for 24 h. The EC_50_ for STS on RIN-m5F beta-cells was determined by their cytotoxicities.

When RIN-m5F cells were treated with 0.1–0.6 μM STS, the cytotoxicity of 0.1 and 0.2 μM STS treatments dramatically increased from 34 to 48% while those of 0.3 to 0.6 μM treatments still increased but only marginally (**Figure 2A**). From the dose response curve, an EC_50_ value of 0.123 μM was obtained, which is lower than previously reported ∼0.5 μM for NS-1-derived 832/13 pancreatic cells (Collier et al., 2011), indicating that different pancreatic beta-cell lines respond to STS treatment differently.

Under apoptotic conditions, both caspase-3 and MST1 are known to be activated (Jacobson et al., 1996; Ardestani et al., 2014). Therefore, we performed western blotting to monitor the activation of caspase-3 and MST1 in RIN-m5F cells after incubated with increasing concentrations of STS. Results showed that the band intensities of both pro-caspase-3 (35 kD) and MST1 (60 kD) decreased while those of their cleaved forms (17 kD for caspase 3 and 34 kD for MST1) increased in a dose-dependent manner under 0.1–0.6 μM STS treatments (**Figure 2B**), indicating that both caspase-3 and MST1 were activated by STS treatments. The cytotoxicity results together with western blotting results suggest that the STS-induced apoptosis RIN-m5F cell-based model could be used to study the cytoprotective effects of asialo-rhuEPO^P^. The EC_50_ of 0.123 μM derived from the dose response curve (**Figure 2A**) was used for studying the cytoprotection of asialo-rhuEPO^P^ and its protective mechanism.

### Asialo-rhuEPO^P^ Protected Pancreatic Beta-cells against STS-induced Cell Injury

**FIGURE 3:**
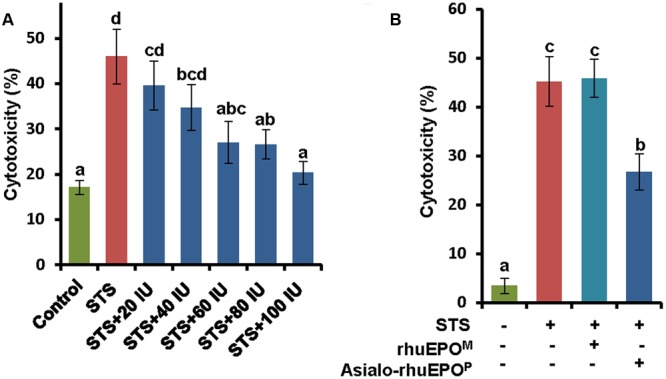
**Cytoprotective effects of asialo-rhuEPO^P^ against STS-induced apoptosis in RIN-m5F cells. (A)** Cytoprotective effects of 20, 40, 60, 80, and 100 IU asialo-rhuEPO^P^ against 0.123 μM STS-induced cell death. **(B)** Cytoprotective effects of 60 IU asialo-rhuEPO^P^ and rhuEPO^M^ against 0.123 μM STS-induced cell death. Data represent the average ± SE. Different letters labeled represent significant difference at *p*<0.05 level.

After confirming STS-induced toxicity in the RIN-m5F beta-cell model, the protective effect of asialo-rhuEPO^P^ against STS-induced cell injury was studied using this cell line. Cells were simultaneously treated with 0.123 μM STS alone, or 0.123 μM STS with 20, 40, 60, 80, or 100 IU/ml asialo-rhuEPO^P^ for 24 h; and the LDH assay was used to determine the cytotoxicity. STS alone treatment resulted in 46% cytotoxicity whereas simultaneous treatments with STS and 20-100 IU of asialo-rhuEPO^P^ resulted in lesser cytotoxicities (values ranging from 40 to 20%) (**Figure 3A**). The cytoprotection rates were then computed from cytotoxicities. Compared to the STS alone treatment, simultaneous treatment with STS and 20, 40, 60, 80, or 100 IU/ml asialo-rhuEPO^P^ gave cytoprotection rates of 14, 25, 41, 42, and 56%, respectively. These results indicate that asialo-rhuEPO^P^ protects pancreatic beta-cells against STS-induced cell injury. Since there was only a marginal increase in protective effect of asialo-rhuEPO^P^ above 60 IU, and considering the cost of the recombinant EPO protein, we chose 60 IU/ml as an optimal concentration, which was used for its protective mechanism study as well as the comparison study with rhuEPO^M^.

To compare cytoprotective effects of asialo-rhuEPO^P^ with rhuEPO^M^, the concentration of 60 IU/ml was used for both types of rhuEPOs. The results showed that asialo-rhuEPO^P^ conferred 41% cytoprotection by comparing cytotoxicity of STS+asialo-rhuEPO^P^ treatment to that of STS alone whereas rhuEPO^M^ did not show any protection (**Figure 3B**). The protective effect of asialo-rhuEPO^P^ is consistent with the observed result shown in **Figure 3A**. In order to understand why rhuEPO^M^ did not exhibit cytoprotection in our experimental system, we measured rhuEPO^M^ and asialo-rhuEPO^P^ after 24 h in the spent medium by ELISA. The purpose was to determine how much of the rhuEPO^M^ was used up by RIN-m5F cells (via binding to the EPOR, receptor internalization and degradation) in comparison to asialo-rhuEPO^P^. The results showed that added rhuEPO^M^ was still in medium with only 7% reduction while added asialo-rhuEPO^P^ was reduced 70% (data not shown), suggesting that only tiny amount of rhuEPO^M^ was used by the RIN-m5F cells. This scenario can result from differences in the affinity of rhuEPO^M^ and asialo-rhuEPO for the cytoprotective receptors. Indeed, asialo-rhuEPO has been shown to have higher affinity for EPOR than rhuEPO^M^ (Imai et al., 1990; Dong et al., 1992).

### Anti-apoptotic Effect of Asialo-rhuEPO^P^ in Pancreatic Beta-cells is Through Suppressing MST1 and Caspase-3 Activation

**FIGURE 4:**
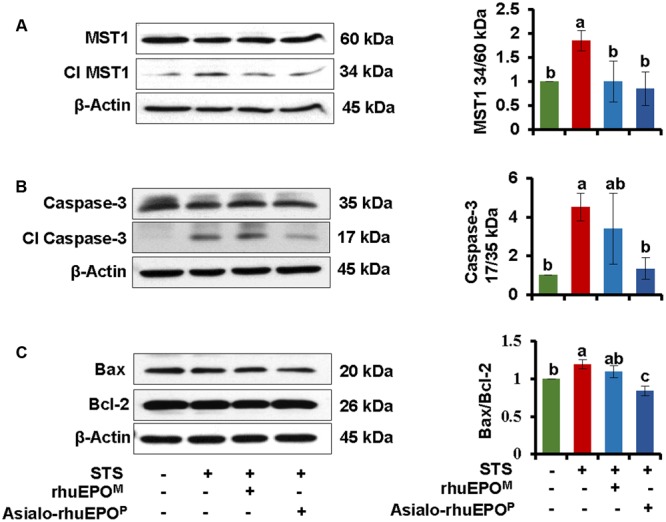
**Western blot of MST1 (A)**, Caspase-3 **(B)**, Bax and Bcl-2 **(C)**. The levels of these proteins were measured in cell lysates prepared from cells treated with PBS containing 0.1% BSA (vehicle control), 0.123 μM STS, 0.123 μM STS+60 IU/ml rhuEPO^M^ or 0.123 μM STS+60 IU/ml asialo-rhuEPO^P^. Active MST1 and caspase-3 were detected using an anti-MST1 and anti-caspase-3 antibody, respectively, which also cross-react with proMST1 and procaspase-3. Bax and Bcl-2 specific antibodies were used to detect these proteins. β-Actin was used as internal control. The experiment was repeated twice. All data plotted are the average of two independent experiments ± SD. Different letters labeled represent significant difference at *p* < 0.05 level.

To understand the cytoprotective mechanism of asialo-rhuEPO^P^ against STS-induced apoptosis in pancreatic beta-cells, we first determined whether its anti-apoptotic function is via suppression of the activation of MST1 and caspase-3. Both inactive MST1 and pro-caspase-3 and their active forms were analyzed by western blotting in beta-cells collected from vehicle control, STS alone, STS+rhuEPO^M^ or STS+asialo-rhuEPO^P^ treatment. The results showed that STS alone could significantly activate both MST1 and caspase-3 with an increase of 1.9-fold of MST1 34 kD/60 kD ratio (**Figure 4A**) and 4.5-fold increase of caspase-3 17 kD/35 kD ratio (**Figure 4B**) compared to those of the vehicle control. In STS+asialo-rhuEPO^P^ treated group, the intensities of cleaved MST1 and cleaved caspase-3 bands were dramatically reduced compared to corresponding bands in STS-treated alone cells. STS+rhuEPO^M^ treatment has similar MST1 34 kD/60 kD ratio as STS+asialo-rhuEPO^P^ treatment, but has 2.5-fold higher caspase-3 17 kD/35 kD ratio than the latter one. The above results indicate that the cytoprotection of asialo-rhuEPO^P^ occurs through suppressing MST1 and caspase-3 activation in beta-cells.

A previous study found that MST1-induced apoptosis proceeds via the mitochondrial-dependent pathway by inducing pro-apoptotic Bax and inhibiting anti-apoptotic Bcl-2, which results in alteration of Bax/Bcl-2 ratio (Ardestani et al., 2014). A higher Bax/Bcl2 ratio promotes apoptosis, whereas a lower ratio promotes cell survival (Korsmeyer et al., 1993). Therefore, the expression levels of mitochondrial proteins Bax and Bcl2 were analyzed. Western blot results showed that pro-apoptotic Bax was reduced while anti-apoptotic Bcl-2 was increased in cells treated with STS+asialo-rhuEPO^P^ compared to those of cells STS alone treated cells (**Figure 4A**). The Bax/Bcl-2 ratio of STS+asialo-rhuEPO^P^ was significantly reduced (29%). For STS+rhuEPO^M^ treatment, the same pattern of reduced expression of Bax and increased expression of Bcl-2 was also observed compared to STS alone treatment. However, the reduction in Bax/Bcl2 ratio was much smaller than that of STS+asialo-rhuEPO^P^ treatment, further supporting that asialo-rhuEPO^P^ not only has cytoprotective function but also has better cytoprotective effect than rhuEPO^M^.

### Anti-apoptotic Effect of Asialo-rhuEPO^P^ in Pancreatic Beta-cells is Through Activating AKT

**FIGURE 5:**
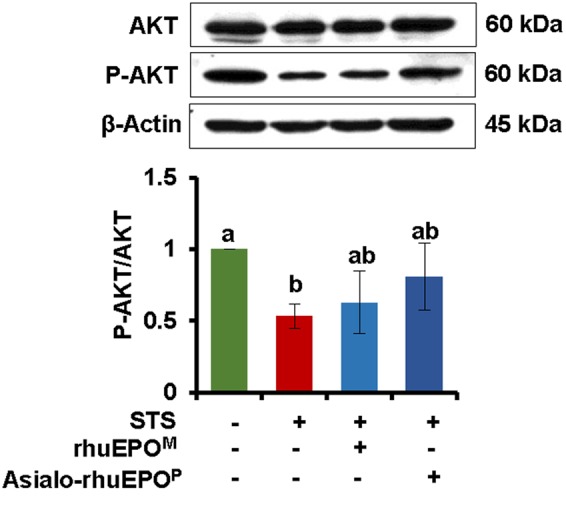
**Western blot of AKT.** The ratio of AKT and p-AKT were measured in cell lysates prepared from cells treated with PBS containing 0.1% BSA (vehicle control), 0.123 μM STS, 0.123 μM STS+60 IU/ml rhuEPO^M^ or 0.123 μM STS+60 IU/ml asialo-rhuEPO^P^. For detection of p-AKT and AKT, the blot was probed with anti-p-AKT antibody first followed by stripping the blot and re-probing with anti-total AKT antibody. The experiment was repeated twice. All data plotted are the average of two independent experiments ± SD. Different letters labeled represent significant difference at *p* < 0.05 level.

It has been reported that the EPOR is present in pancreatic islets (Fenjves et al., 2003; Choi et al., 2010), which is required by rhuEPO-mediated cytoprotection in beta-cells (Choi et al., 2010) as well in other non-hematopoeitic cells (Brines and Cerami, 2006; Hand and Brines, 2011; Broxmeyer, 2013). RhuEPO-mediated cytoprotective signaling in beta-cells is shown to occur via the activation of JAK2 and downstream PI3K/AKT pathway (Choi et al., 2010). Thus, the phosphorylation levels of AKT were analyzed by western blotting in above mentioned four types of treated cells. The results showed that STS treatment significantly inhibited AKT phosphorylation with 47% reduction in p-AKT/AKT ratio (**Figure 5**). Both asialo-rhuEPO^P^ and rhuEPO^M^ treatments partially restored AKT phosphorylation, but the effect was more pronounced in the former than the latter one. The result indicates that the antiapoptotic effect of asialo-rhuEPO^P^ is established by preserving AKT phosphorylation.

### Asialo-rhuEPO^P^ Prevented PDX1 Degradation and Restored Insulin Secretion

It has been shown that MST1 regulates PDX1 at the post-transcriptional level leading to ubiquitination and degradation (Ardestani et al., 2014). Reduced expression of PDX1 can affect not only beta-cell survival but also insulin production (Brissova et al., 2002; Johnson et al., 2003). Therefore, the expression levels of PDX1 were measured in the above mentioned four types of treated RIN-m5F cells. Correspondingly, the levels of secreted insulin in the culture media were also measured.

**FIGURE 6:**
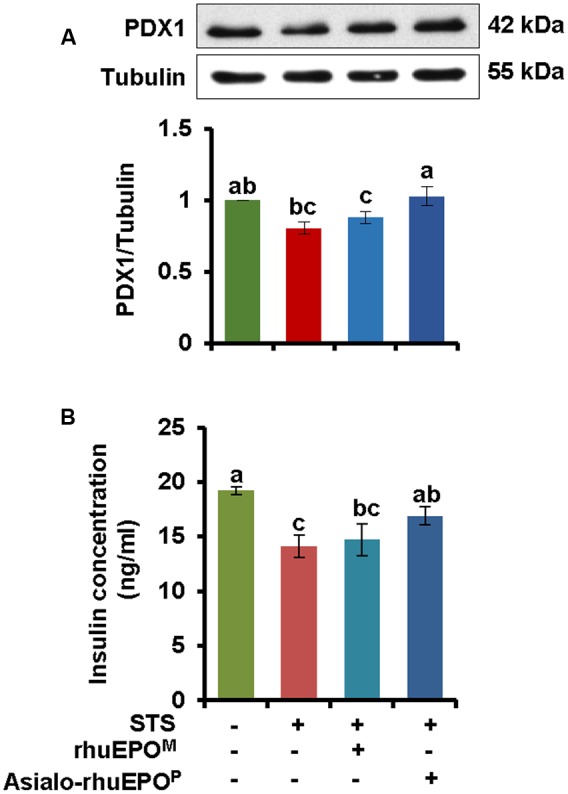
**Western blot of PDX1 (A)** and the levels of secreted insulin in cultured medium **(B)**. **(A)** The levels of PDX1 protein were measured in cell lysates prepared from cells treated with PBS containing 0.1% BSA (vehicle control), 0.123 μM STS, 0.123 μM STS+60 IU/ml rhuEPO^M^, or 0.123 μM STS+60 IU/ml asialo-rhuEPO^P^. **(B)** Secreted insulin in cultured medium. Secreted insulin levels were measured in media from above four cultures. The measurement was repeated three times. The experiment was repeated twice. All data plotted are the average of two independent experiments ± SD. Different letters labeled represent significant difference at *p* < 0.05 level.

Compared to vehicle control cells, the level of PDX1 was reduced approximately 20% in STS-treated cells (**Figure 6A**). When the cells were treated with STS+asialo-rhuEPO^P^ or STS+rhuEPO^M^, the level of PDX1 protein remained higher than in STS alone treated cells (**Figure 6A**). It was noticed that asialo-rhuEPO^P^ treatment was able to restore PDX1 protein level to the vehicle control levels while rhuEPO^M^ treatment could restore 9% compared to STS alone treatment. These results further indicate that asialo-rhuEPO^P^ protected the STS-induced PDX1 degradation and conveyed better protective effect than rhuEPO^M^.

When secreted insulin levels in spent medium were measured, its concentrations in the vehicle control, STS alone, STS+rhuEPO^M^ and STS+asialo-rhuEPO^P^ were 18.5, 12.9, 13.5, and 15.8 ng/ml, respectively. STS treatment caused a significant reduction of secreted insulin level (30%) compared to the vehicle control. This result is consistent with the previous report by Akiyoshi and Nakaya (2000) that STS inhibited 37% of secreted insulin in HIT-T15 cells. With asialo-rhuEPO^P^, the secreted insulin level was restored to about 22% compared to STS alone treatment. However, rhuEPO^M^ only provided limited protection with only 4% restoration of the secreted insulin. Consistent with observed PDX1 levels, asialo-rhuEPO^P^, but not rhuEPO^M^, could improve beta-cell function impaired by STS treatment.

## Discussion

Exploring new therapeutics to prevent apoptosis and subsequent dysfunction in pancreatic beta-cells is critical for diabetes treatment. MST1 was found to be a key mediator of apoptotic signaling in beta-cells and has been suggested as a novel target for the discovery of new drugs for diabetes (Ardestani et al., 2014). Recent findings by Fan et al. (2016) demonstrated that blocking MST1/2 kinase activities with a newly identified compound, 4-((5,10-dimethyl-6-oxo-6,10-dihydro-5H-pyrimido[5,4-b]thieno [3,2-e][1,4]diazepin-2-l)amino)benzenesulfonamide (XMU-MP-1), has benefits for intestinal and liver repair and regeneration in mice (Fan et al., 2016). In the present study, we used a STS-induced apoptotic pancreatic beta-cell model to demonstrate that asialo-rhuEPO^P^ could suppress the activation of MST1 as well as caspase-3, displaying its cytoprotective property (**Figures 3, 4**), and that its anti-apoptotic effect could improve impaired insulin secretion through stabilizing PDX1 (**Figure 6**). Our results not only proved that asialo-rhuEPO^P^ is cytoprotective toward beta-cells but also revealed that it can also suppress activation of MST1, a key player that determines the susceptibility of beta-cells to apoptosis and ultimately leading to diabetes. These findings set the stage for future cytoprotective studies in other cell-based diabetic models as well as animal models of diabetes to determine whether it could be developed as a novel agent to treat diabetes.

Previous studies have shown that rhuEPO has protective effects on beta-cells in streptozotocin-induced experimental diabetic mice (Choi et al., 2010) and rats (Chen et al., 2015). RhuEPO was also found to protect pancreatic NIT-1 beta-cells against cytokines (IL-1β, TNF-α, and IFN-γ)-induced apoptosis (Shuai et al., 2011). Conversely, it is surprising that rhuEPO^M^ showed no protection in the current STS-induced apoptotic beta-cell model whereas asialo-rhuEPO^P^ displayed clear cytoprotective effects (**Figure 2B**). Since we found that rhuEPO^M^ was still in the spent medium and not degraded during the treatment process, lack of protective effect of rhuEPO^M^ could relate to either the STS-induced apoptotic model employed or to the differences in the affinity of rhuEPO and asialo-rhuEPO for the receptor (see below). STS is a prototypical ATP-competitive kinase inhibitor, which can bind to many kinases with high affinity (Karaman et al., 2008). In the case of rhuEPO-mediated cytoprotection, it has been reported to act at multiple levels, including not only inhibition of apoptosis (Digicaylioglu and Lipton, 2001) but also reduction of reactive oxygen species/glutamate (Kawakami et al., 2001), modulation of inflammation (Kawakami et al., 2001) and recruitment of stem cells (Shingo et al., 2001). In erythroid progenitor cells, binding of EPO to its receptor is known to initiate signaling cascades, including PI3K, STAT5 and Ras/MAPK pathways, which ultimately result in their survival, proliferation, and differentiation (Neumann et al., 1993; Sulahian et al., 2009). It is unknown at this time which kinase(s) in these signaling pathways is affected. Furthermore, asialo-rhuEPO (lacking terminal sialic acid residues) has higher affinity for EPOR than rhuEPO^M^ bearing terminal sialic acid residues (Imai et al., 1990; Dong et al., 1992). It also remains elusive as discussed in the following paragraph whether asialo-rhuEPO^P^ and rhuEPO^M^ share the same cytoprotective mechanism(s). Further studies are needed to understand why rhuEPO^M^ failed to provide protection to RIN-m5F cells under the STS-induced apoptosis. Nevertheless, asialo-rhuEPO^P^ was found to have cytoprotective function. Once its cytoprotective function can be confirmed *in vivo*, all advantages related to the plant-based expression to produce asialo-rhuEPO^P^, such as the low cost of production, freedom from human pathogen, lack of hematopoietic activity-related side effects and ease of scale-up, can be exploited to develop a potentially inexpensive and safe beta-cell protective agent.

Concerning the protective mechanism of asialo-rhuEPO^P^, although all players involved in the cell survival pathway were not investigated in the present study, there is a good reason to believe that the activation of PI3K/AKT pathway was responsible for observed cytoprotective effects of asialo-rhuEPO^P^. AKT is a well-known MST1 inhibitor (Yuan et al., 2010). Yuan et al. (2010) reported that AKT interacts with MST1 resulting in phosphorylation of the conserved Thr^120^ residue in MST1 thereby inhibiting its kinase activity, translocation to the nucleus and autophosphorylation of its Thr^183^residue. These authors further reported that phosphorylation of MST1 by AKT reduces caspase-3 activity. The PI3K/AKT pathway has also been shown to play a critical role in glucose metabolism and insulin signaling. Insulin signaling via AKT was shown to promote translocation of glucose transporter type 4 through activation of AS160 thereby increasing glucose uptake in mice fed high fat high sucrose diet (Collino et al., 2014). Besides the PI3K/AKT pathway, EPO-mediated protection was also found to be tied to several other signaling pathways, such as, the mTOR, Wnt, and WISP1 signaling, FoxO, (SIRT1; *Saccharomyces cerevisiae*), and AMPK (Maiese, 2015). However, further studies are needed in order to fully understand the mechanism of cytoprotection afforded by asialo-rhuEPO^P^ to pancreatic beta cells.

Since the *N*-glycan chains on rhuEPO and asialo-rhuEPO are different (**Figure 1**), these proteins differ with respect to their binding affinities to the receptor. RhuEPO is a negatively charged molecule because of terminal sialic acid residues, whereas asialo-rhuEPO is a basic protein (Kittur et al., 2015). Sialylation has been shown to have negative effect on receptor binding in which rhuEPO exhibits much less affinity for EPOR than asialo-rhuEPO (Imai et al., 1990; Dong et al., 1992; Darling et al., 2002). Furthermore, it was found that the removal of negatively charged sialic acid residues resulted in marked enhancement in the *k*_on_ but maintained the same *k*_off_ (Darling et al., 2002). This enhancement in the *k*_on_ of asialo-rhuEPO has been suggested to transduce a strong cell survival signal (Imai et al., 1990). This may explain the different cytoprotective effects observed in rhuEPO^M^ and asialo-rhuEPO^P^ (**Figure 3**). Our previous study in neuronal-like cells N2A (Kittur et al., 2013) and recent study in HL-1 murine cardiomyocytes (F. Kittur et al., unpublished data) both found that the asialo-rhuEPO^P^ has ∼2-fold better protective effects than rhuEPO^M^ against STS-induced apoptosis. These results also imply that rhuEPO^M^ and asialo-rhuEPO^P^ could have different binding efficacy to the receptor. Future studies are warranted to answer whether rhuEPO^M^ and asialo-rhuEPO^P^ use the same receptor with different binding efficiency or even bind to different receptor to influence downstream signaling pathways.

In summary, our results demonstrate that asialo-rhuEPO^P^ provides up to 48% cytoprotection to the RIN-m5F beta-cell line against STS-induced cell death, and that its cytoprotection mechanism involves the reduction in MST1 activation, interrupting the apoptotic process and enhancing insulin secretion. The current study leads us to carry out future cytoprotective studies to conclude whether asialo-rhuEPO^P^ can be used as a drug for diabetes or even as a broad tissue/cell-protective agent for other diseases.

## Author Contributions

JX, EA, FK, and DS conceived and designed the experiments; EA, FK, and YL performed the experiments; EA, FK, C-YH, JX, and DS analyzed the data; EA, FK, JX wrote the article with contributions of all the authors.

## Conflict of Interest Statement

JX, FK, C-YH are inventors of filed patent “Methods for the production of cytoprotective asialo-erythropoietin in plants and its purification from plant tissues” (PCT NUMBER: US2013031382, pending). The other authors declare that the research was conducted in the absence of any commercial or financial relationships that could be construed as a potential conflict of interest.

## Abbreviations

AMPK: AMP activated protein kinase
Asialo-rhuEPO: asialo-erythropoietin
Asialo-rhuEPO^P^: plant-produced asialo-rhuEPO
BSA: bovine serum albumin
β-CR: β-common receptor
EPO: erythropoietin
*EPO*: human erythropoietin gene
EPOR: EPO receptor
FoxO: mammalian forkhead transcription factors of the O class
*GalT*: human β1,4-galactosyltransferase gene
*k*_off_: off-rate
*k*_on_: associate rate constant
LDH: lactate dehydrogenase
MST1: Mammalian Sterile-20-like Kinase
mTOR: mechanistic target of rapamycin
PBST: phosphate-buffered saline containing 0.1 % Tween-20
PDX1: pancreatic duodenal homeobox-1
rhuEPO: recombinant human erythropoietin
rhuEPO^M^: mammalian cell-produced rhuEPO
SIRT1: silent mating type information regulation 2 homolog 1
STS: staurosporine
